# Autophagy Suppresses Invasiveness of Endometrial Cells through Reduction of Fascin-1

**DOI:** 10.1155/2018/8615435

**Published:** 2018-06-11

**Authors:** Xiaomei Luo, Wei Cheng, Shizhang Wang, Zhihong Chen, Jieqiong Tan

**Affiliations:** ^1^Department of Obstetrics and Gynecology, The Maternal and Child Healthcare Hospital of Hunan Province, Changsha 410005, China; ^2^Department of Pathology, The People's Hospital of Hunan Province, Changsha 410005, China; ^3^Center for Medical Genetics, School of Life Sciences, Central South University, Changsha 410078, China

## Abstract

**Objective:**

Autophagy has been reported to be involved in the development of various disorders such as neurodegenerative and metabolic diseases and tumors. Autophagy activators and inhibitors are also potential therapeutics for these diseases. However, the mechanism of autophagic involvement in different diseases is not the same, and the role of autophagy in endometriosis (EM) has not yet been elucidated. This research investigated the mechanism by which autophagy acts in EM, with the aim of establishing a theoretical basis for its prevention and treatment through the targeted interference with autophagy.

**Methods:**

We used an RNA interference fragment targeting ATG5, the autophagy activator rapamycin, and the autophagy inhibitor 3-MA or overexpression of filopodia-related protein fascin-1, in conjunction with clonogenic assays, growth curves, and scratch assay to investigate the influence of autophagy on cellular growth, proliferation, and invasiveness. We collected specimens from 20 clinical cases of EM and investigated the protein expression of the autophagic marker LC3-II, the autophagic substrate p62, and fascin-1.

**Results:**

Rapamycin was able to inhibit the proliferation and colony formation of the endometriotic cell line CRL-7566, whereas the autophagy inhibitor 3-MA as well as the interference with the autophagy-related gene ATG5 had the opposite effect. More importantly, the autophagy activator rapamycin was able to inhibit the growth of filopodia in the endometriotic cells, and the overexpression of the fascin-1 restored the rapamycin-induced decrease of invasiveness. We found that the expression of the autophagy marker LC3-II was significantly reduced among the clinical EM specimens compared to the control group, while the expressions of fascin-1 and autophagic substrate p62 were increased.

**Conclusion:**

Our results indicate that the inhibition of autophagy and exogenous expression of fascin-1 may promote the invasiveness of endometrial cells. As a corollary, autophagy represents a potential target for the treatment of EM.

## 1. Introduction

Endometriosis (EM) denotes the occurrence, growth, and infiltration of glandular and mesenchymal endometrial tissue outside the endometrium and is often characterized by repeated bleeding or nodule formation [1]. It is a frequently encountered gynecological endocrine disease and has a high incidence [1]. Its accompanying symptoms, which include the inability to conceive and chronic pain, severely impact the reproductive health and quality of life in women of childbearing age. The incidence of EM in women in this age group is as high as 15%, and among patients struggling with infertility, it can be as high as 50% [2]. The major theories surrounding the causes of EM include the counterflow of menstrual blood, metaplasia of the coelomic epithelium, abnormal implantation, and the dissemination of blood and lymph, as well as immunological explanations [3]. Nevertheless, the molecular mechanism of its pathogenesis is not completely clear. Recent research has shown that autophagy plays an inordinately important role in the emergence and progression of numerous human illnesses, such as neurodegenerative disease, tumors, infections, immunological disorders, and cardiovascular disease [4, 5]. Autophagy is one of the major ways in which eukaryotic cells remove abnormal proteins and damaged organelles through lysosomal degradation [6]. It plays a crucial role in maintaining cellular homeostasis and balancing the energy metabolism, as well as in cellular responses to various external and internal stimuli [7, 8]. Moreover, an increasing amount of evidence shows that autophagy also plays important roles in the occurrence and progression of neoplasms, as well as the regulation of various biological behaviors of cells [9]. During the early stages of tumor development, the inhibition of autophagic activity can induce precancerous cells to continue growing, which means that autophagy has an antitumor function at this stage [10]. By contrast, the cells of advanced tumors utilize autophagy to counteract the lack of nutrients and blood supply, giving autophagy a tumor-promoting function at this stage [11, 12]. Furthermore, it has also been discovered that autophagy can protect tumor cells from damage during radiotherapy [13, 14]. Accordingly, the role of autophagy in tumor biology is relatively complex, with different involvement in pathological processes at different times [15]. Although some studies [3, 16, 17] have reported that the level of autophagy may have a relationship with the occurrence of EM, the exact role and mechanism of its involvement have not yet been elucidated.

## 2. Materials and Methods

### 2.1. Cells, Plasmids, and Antibodies

The endometrial cell line CRL-7566 (ATCC) was purchased from the American Type Culture Collection, Manassas, Virginia, USA. Antibodies reactive against LC3-II, P62, actin, and ATG5 were purchased from Cell Signaling Technology (Danvers, USA). Antibodies against fascin-1 were from Abcam (Cambridge, USA). Antibodies against FLAG-tagged proteins were from Sigma-Aldrich, USA. The plasmid encoding FLAG-fascin-1 was purchased from Addgene (Cambridge, USA), and the ATG5 siRNA interference fragment was synthesized by Genepharma (Shanghai, China).

### 2.2. Reagents

Rapamycin, 3-MA, and MTT were purchased from Sigma-Aldrich, USA. Lipofectamine 2000 was from Invitrogen (San Diego, USA). All other reagents were from Shanghai Shengwu Huaxue Company, China.

### 2.3. Cell Culture and Transfection

The CRL-7566 cells were cultured in high-glucose DMEM complete medium (Invitrogen, San Diego, USA) with the addition of 5% fetal bovine serum (FBS; Invitrogen, San Diego, USA), in an atmosphere comprising 5% CO_2_ at 37°C. Plasmid transfection and siRNA interference were conducted using Lipofectamine 2000 (Invitrogen, San Diego, USA), according to the manufacturer's instructions.

Briefly, 0.5 *μ*g of plasmid DNA or 5*μ*M siRNA was added to 50 *μ*L of Opti-MEM (Invitrogen, San Diego, USA) and mixed well. At the same time, 2*μ*L of Lipofectamine 200 was mixed with 50*μ*L of Opti-MEM, mixed, and incubated statically at room temperature for 5min. After the two solutions were mixed and incubated statically at room temperature for 15min, the resulting equilibrated solution was added to each well of a 24-well plate. Following 12h of incubation, the medium was exchanged for fresh one, and the cells were harvested after 36h for further experiments. The interference segment targeting ATG5 had the following sequence: 5'- AAGCAACTCTGGATGGGATTG-3'.

### 2.4. Immunofluorescence

The endometrial CRL-7566 cells were seeded at approx. 1×10^5^ cells/well into the wells of a 24-well plate with sterilized round glass coverslips and cultured in DMEM with 10% FBS at 37% in an atmosphere comprising 5% CO_2_. After the culture medium was removed, the cells were fixed with 4% paraformaldehyde/30% sucrose/PBS for 15min at 25°C, washed with PBS for 5min two times, permeabilized with 0.1% Triton X-100/PBS for 10min at 25°C, washed with PBS for 10min two times, blocked with 5% BSA in PBS for 30min at 25°C, incubated with the primary antibody diluted as 1:1000 in 5% BSA/PBS at room temperature for 1h, washed with PBS for 5min five times, blocked with 5% BSA/PBS for 20min at 25°C, incubated with the secondary antibody diluted as 1:10000 in 5% BSA/PBS at room temperature for 1h, washed with PBS for 5min seven times, stained for 2min with 1 *μ*g/mL DAPI, washed with PBS for 5min two times, and finally washed once with water. A drop comprising 4*μ*L of 60% glycerol was placed on top of the glass slide with fixed stained cells, and the glass coverslip was gently lowered on top of the glass slide from one side to the other. The expression of proteins was examined using a confocal laser scanning microscope and photographed for documentation purposes. Filopodia number and length were measured using ImageJ 1.46r. All filopodia within a 100*μ*m region at the leading edge of NC stream 1 were included in analyses. Over 300 filopodia were manually counted for each condition from three independent experiments.

### 2.5. Immunoblotting

The cells were lysed using 2×SDS sample buffer for 10min at 25°C and ultrasonicated for 5s three times. The BCA protein assay kit was used to determine the protein concentration, using a sample amount of 20*μ*g total protein/well. According to the molecular cloning methods, an 8% SDS-page gel with a 5% stacking gel was prepared, and protein separation was conducted under constant voltage of 80V. The resulting protein bands were electroblotted at 290mA for 2h onto PVDF membrane (Millipore, USA) and blocked with 5% BSA in PBS at 25°C for 1h. The blocked membrane was incubated with the primary antibody diluted as 1:1000 in 5% BSA/PBS for 1h at 25°C, washed with 0.1% Triton X-100/PBS for 10 min two times, incubated with the secondary antibody diluted as 1:10000 in 5% BSA/PBS for 1h at 25°C, washed with 0.1% Triton X-100/PBS for 10 min three times, incubated for 5min at 25°C with the ECL reagent prepared according to the manufacturer's instructions, and finally developed in the darkroom.

### 2.6. Determination of Growth Curves of the Cells

A 6-cm diameter culture dish was seeded with 1×10^5^ cells; 200*μ*g/mL of G418 was added to maintain the cultures and the cells cultured at 37°C in an atmosphere comprising 5% CO2 for 72h. Individual dishes were sampled in intervals of 24h, digested with 0.25% trypsin at 37°C for 3min, neutralized with serum-containing medium, and agitated to yield a single-cell suspension. Subsequently, 0.5mL trypan blue solution, 0.3mL HBSS, and 0.2mL cell suspension were mixed and subsequently incubated at room temperature for 10min. For cell staining, the trypan blue solution was dripped onto the cell-counting plate, and the number of live cells was determined at 24, 48, and 72 h after transfection. Each sample was quantified 5 times and the average value was used as the result. The experiments were done in triplicate independently. GraphPad Prism 5 was used to draw the growth curves and conduct the statistical analysis.

### 2.7. Clonogenic Assay

After transfected cells were trypsinized routinely, they were resuspended in complete medium and agitated to yield a single-cell suspension. The resulting suspension revealed a single-cell ratio exceeding 95% upon microscopic inspection. Subsequently, the cells were counted on a hemocytometry glass slide, and the cell concentration was adjusted with complete medium to yield a defined dilution ratio. Using a dilution that corresponds to 500 cells per Petri dish, 10-cm diameter dishes were seeded with 12mL of the cell suspension and swayed to thoroughly mix the cells. The thus seeded culture dishes were incubated at 37°C in an atmosphere comprising 5% CO_2_ for 14 days, with medium changes every three days. The medium was supplemented with 200*μ*g/mL G418 to maintain the cultures. When colonies visible to the naked eye appeared inside the culture dishes, the culture medium was removed, and the cultures were carefully washed with 1×PBS three times, after which the cells were fixed with 4% paraformaldehyde for 15min at 25°C and dried in the open air. The dried colonies were stained with Giemsa staining solution for 10 min, washed with water, and dried. Finally, all colonies in each 100-mm culture dish were counted, and the results were used for statistical analysis.

### 2.8. Scratch Assay

The cells were cultured to 90% confluency in six-well plates; then a thin scratch (wound) was made in the central area using a 10-ml pipette tip. Detached and damaged cells were carefully removed with PBS and the medium was replaced with serum-free medium. Wound closure was observed by light microscopy and images were captured after 24h.

### 2.9. Collection of Clinical Cases

Pathological samples and normal endometrial tissue from 20 cases of EM were collected according to the ethical requirements of Hunan Province People's Hospital, Changsha, China, and signed informed consent statements were collected from all study participants. Detailed clinical manifestations, including pathological indices, status of metastasis, recurrence, and follow-up, were collected from all patients. Normal eutopic endometrial tissues were obtained from premenopausal women undergoing hysterectomy for uterine leiomyoma. Ectopic endometriotic tissues were obtained from ovarian endometriotic cysts. A total of 20 patients had histological and laparoscopic evidence of advanced stage endometriosis (stage III or IV) and had regular menstrual cycles. Study participant had not received any hormonal therapy during the previous 3 months. All tissues were classified into five categories according to day of menstrual cycle as early proliferative (days 4-7), mid proliferative (days 8-10), late proliferative (days 11-14), early secretory (days 15-18), mid secretory (days 19-22), or late secretory (days 23-28) phases. Menstrual cycle day was established based on the patient's menstrual history. The average age of participants was 38.2+4.6 years for eutopic endometrial tissues and 29.3+6.6 years for ectopic endometrial tissues. Of the 20 eutopic and 20 ectopic endometrial samples, four cases were each in early proliferative, late proliferative, early secretory, mid-secretory, and late secretory phases.

### 2.10. Determination of Protein Levels in Tissue Samples

After the tissue was homogenized using 0.1% Triton X-100 in PBS, an equal volume of 2×SDS sample buffer was added to each sample, lysed for 10min at 25°C, ultrasonicated for 5s three times, and used in the same way as described in the Immunoblotting.

### 2.11. Statistical Analysis

The data were analyzed using GraphPad Prism 5 software. Analysis of variance (ANOVA) and Tukey's test (for within-group two-by-two comparisons) or Dunnett's multiple comparison test (for comparisons of each group with the control group) was used. Differences with* p* values <0.05 were considered to be statistically significant.

## 3. Results

### 3.1. Interference with the Autophagy Level of Endometrial CRL-7566 Cells

**Figure 1:**
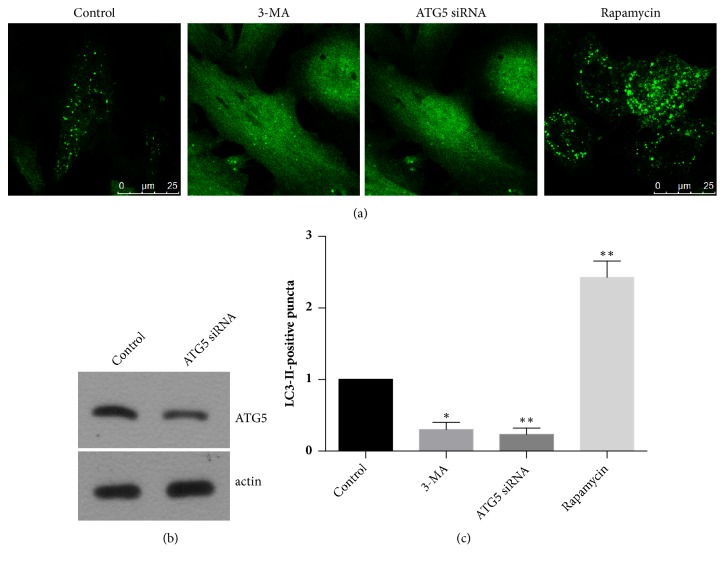
Interference with the autophagy level of endometrial CRL-7566 cells. (a) Immunofluorescence showing the influence of each of the interference methods on the abundance of LC3-positive puncta in endometrial CRL-7566 cells. (b) Immunoblot- determination of the expression level of ATG5 after transfection with an RNAi fragment targeting the corresponding gene. Actin was used as internal control. (c) Statistical analysis of the results obtained from (a).  ^*∗*^*p*<0.05 compared with the control.  ^*∗∗*^*p*<0.01 compared with the control.

We used two different methods to interfere with the autophagy level of endometrial CRL-7566 cells-treatment with the autophagy inhibitor 3-MA and transfection with an RNA interference fragment targeting the ATG5 gene in order to repress autophagy. The results of immunoblotting have shown that the expression level of ATG5 was obviously reduced upon RNA interference (Figure 1(b)). Furthermore, we also used rapamycin to activate autophagy. As can be seen in Figure 1(a), the number of LC3-positive autophagic puncta was obviously decreased in the 3-MA and ATG5 groups, while their number was visibly increased in the rapamycin-treated group. Statistical analysis revealed that the differences in the number of the LC3-positive puncta in each group were significantly increased compared with the control group (Figure 1(c)).

### 3.2. Activation of Autophagy Suppresses the Proliferation and Clonogenicity of CRL-7566 Cells

We used two different methods to interfere with the autophagy level of endometrial CRL-7566 cells—treatment with the autophagy inhibitor 3-MA and transfection with an RNA interference fragment targeting the ATG5 gene in order to repress autophagy.

**Figure 2:**
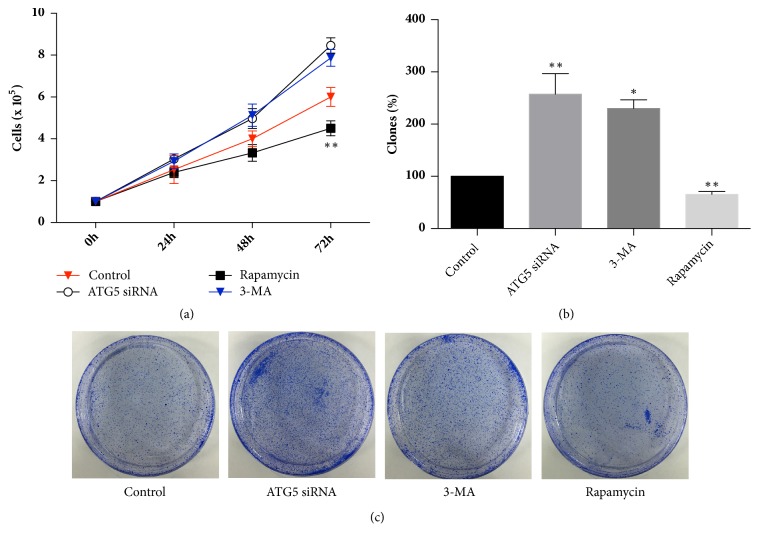
The effect of interference with the autophagy level of endometrial CRL-7566 cells on cell proliferation and clonogenicity. (a) The cell proliferation assay showed that each of the interference methods had an effect on the proliferation of endometrial CRL-7566 cells. (b) The clonogenic assay showed that each of the interference methods had an effect on the clonogenicity of endometrial CRL-7566 cells.  ^*∗*^*p*<0.05 compared with the control.  ^*∗∗*^*p*<0.01 compared with the control. A photograph of Petri-dishes in a representative experiment is shown in (c).

The results of cell proliferation and clonogenic assays showed that the autophagy inhibitor 3-MA and transfection with the RNAi fragment targeting ATG5 were both able to significantly promote the cells' proliferation and clonogenicity, while rapamycin was able to suppress the proliferation and clonogenicity of CRL-7566 cells. Moreover, the differences were all statistically significant (Figure 2).

### 3.3. Activation of Autophagy Suppresses the Formation of Filopodia and Invasiveness of CRL-7566 Cells

**Figure 3:**
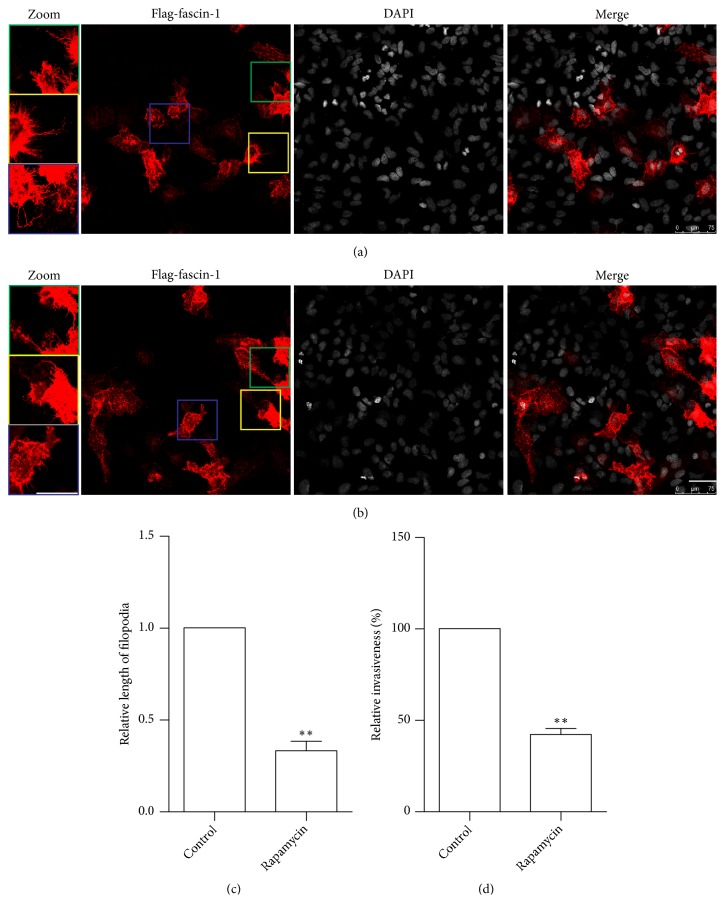
The effects of treatment with the autophagy activator rapamycin on the formation of filopodia and cellular invasiveness. The tagged filopodial marker protein FLAG-fascin was overexpressed to visualize the filopodia (red) and DAPI staining was used to visualize the nuclei (gray). (a) Control treatment (buffer). (b) Rapamycin treatment. (c) Results of the statistical analysis of the relative length of filopodia. The results were normalized to the control group, which was set to 1. (d) Scratch assay analysis of the cells' invasiveness. The results were normalized to the control group, which was set to 100%.  ^*∗∗*^*p*<0.01 compared with the control.

In order to simplify the observation of the cells' filopodia, we overexpressed the tagged filopodial marker protein FLAG-fascin 1, enabling the visualization of filopodia through immunostaining. As can be seen from Figure 3, the filopodia of the rapamycin-treated group (Figure 3(b)) were visibly shorter than those of the nontreated group (Figure 3(a)), and the difference was statistically significant (Figure 3(c)). Additionally, we used the scratch assay to determine the invasiveness of the cells. The results demonstrated that rapamycin treatment reduced the cells' invasiveness (Figure 3(d)).

### 3.4. Fascin-1 Overexpression Reversed the Rapamycin-Induced Inhibition of Cellular Invasiveness

**Figure 4:**
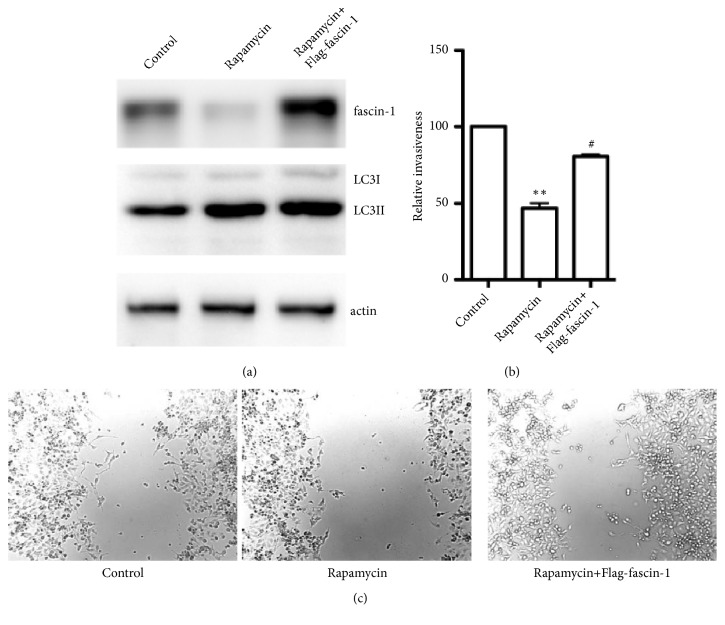
The effect of fascin-1 overexpression on the reduction of cellular invasiveness by rapamycin. (a) Immunoblotting to determine the expression of fascin-1 and LC3-II. Actin was used as internal control. (b) Statistical analysis of the effect of fascin-1 overexpression on the change of cellular invasiveness brought upon by rapamycin treatment.  ^*∗∗*^*p*<0.01 compared with the control;  ^#^*p*<0.05 compared with the rapamycin-treated group. (c) Scratch assay comparing the wound healing properties of treatment indicated (magnification, ×20).

In a further step aimed at investigating the mechanism by which the autophagy activator influences the cells' invasiveness, we measured the influence of rapamycin on the invasiveness of cells overexpressing fascin-1. The results showed that extrinsic expression of fascin-1 did not change the level of autophagy but was nevertheless able to counteract the suppression of cellular invasiveness induced by rapamycin treatment (Figure 4).

### 3.5. The Autophagy Levels of EM Tissues Were Lower Than Those of Controls Comprising Normal Endometrial Tissue

**Figure 5:**
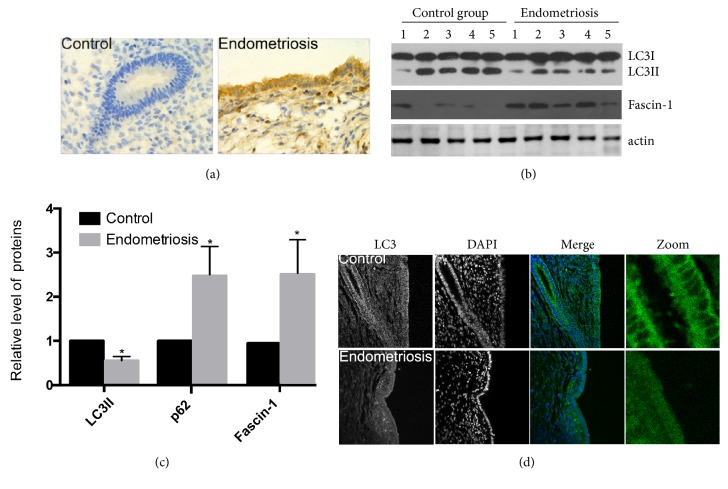
The levels of the autophagy marker LC3-II and the autophagic substrate p62 in endometriotic tissue samples and corresponding normal tissue controls. (a) Immunohistochemistry results showing the protein levels of p62 in EM tissues and controls. (b) Immunoblotting results showing the protein levels of LC3-II in EM tissues and controls. Actin was used as internal control. (c) Statistical analysis of the protein levels of LC3-II and p62 in EM tissues and controls.  ^*∗*^p<0.05 compared with the control. (d) Results of immunofluorescence for the autophagy marker LC3-II (green) and nuclei (DAPI, blue).

We collected pathological samples and normal control tissues from 20 cases of EM and used antibodies against LC3-II and p62 to determine their respective protein levels. LC3-II is an autophagic marker protein, and its reduced levels can indicate a reduced rate of autophagy. By contrast, p62 is a substrate of the autophagic-lysosomal degradation pathway, and an increase of its levels is considered an indicator of suppressed autophagy. Immunohistochemistry and immunostaining result showed the protein levels of p62 in EM tissues are higher than in the controls (Figure 5(a)), while the protein levels of LC3 in EM tissues are lower than in the controls (Figure 5(d)). As can be seen from the example of our results showing the western blot of 5 EM tissue samples and 5 corresponding normal control tissues, the level of the autophagy marker LC3-II was lower in the EM tissue samples than in the controls, while the level of the fascin-1 was increased (Figure 5(b)). The results of statistical analysis shown in Figure 5(c), calculated using data from all 20 cases, show that the levels of LC3-II and p62 in the EM tissue samples had a statistically significant difference to the respective levels in the control tissues.

## 4. Discussion

A large number of recent studies have shown that autophagy plays a significant role in the emergence and development of neoplasms. In fact, changes of autophagic activity have been observed in a large number of tumors, and the expression levels of the autophagy-related protein LC3 differ among different tumors, including a smaller number of reports in brain tumors and lung, cervical, and ovarian cancer [18, 19] and more reports in cancers of the esophagus and the gastrointestinal tract [20, 21]. Endometriosis and malignant neoplasms share a similar mode of growth, invasion, metastasis, and relapse. Accordingly, changes of autophagic activity may also be present in EM, and changes of the level of autophagy may be involved in the emergence and development of EM[17, 22]. Researchers have compared 30 cases of adenomyosis in eutopic endometrium with endometrial tissues from 32 normal women, revealing that the Beclin-1 mRNA and protein expression levels were obviously reduced in the diseased tissues compared to the normal controls [23, 24]. Moreover, Beclin-1 protein expression was negatively correlated with the serum levels of CA-125 and pelvic pain, indicating that there is a certain relationship between autophagy and the occurrence and development of uterine adenomyosis [23, 24]. More importantly, the synthetic progestogen drug dienogest is capable of activating autophagy by suppressing the activity of AKT/mTOR and ERK/mTOR, which in turn induces the apoptosis of endometrial stromal cells [16]. Furthermore, Mullerian inhibiting substance can inhibit the apoptosis of endometrial CRL-7566 cells by activating autophagy [25]. Endometrial cells maintain their proliferative state during the progestational phase by inhibiting autophagy and apoptosis compared to normal tissue. As a corollary, autophagy-activating substances can promote the apoptosis of endometrial cells [26]. In this research, we used two different pathways to interfere with the autophagy levels of endometrial cells and discovered that the low level of autophagy plays a very important role in the maintenance of the growth of endometrial cells. This may be related to the abundant supply of blood and nutrients required by the endometrial cells. Low autophagy levels are correlated with increased expression levels of proteins related to the maintenance and promotion of cellular proliferation. Moreover, we discovered that the autophagy levels in clinical EM tissue samples were significantly lower than in the control group comprising corresponding normal tissues. More importantly, low autophagy levels promoted the invasiveness of endometrial cells. In a further step, we demonstrated that this may be related to the expression of the filopodial protein fascin-1. Fascin-1 is an important component of filopodia—slender and actin-rich protrusions of the cell membrane. Cellular protrusions include the filamentous filopodia, flat lamellipodia, and invasive pseudopodia, among a few other types. Filopodia and lamellipodia play a role in the adhesion during the early stages of tumor invasion. A number of recent studies have shown that fascin-1, which is a crucial component of filopodia, is an important target gene of the Wnt/*β*-catenin signal-transduction pathway. Meta-analysis has shown that the positive expression of fascin-1 in tumor tissues is correlated with a poor prognosis and has a significant relationship with lymphatic metastasis. Moreover, overexpression of fascin-1 can increase the invasiveness and metastasis of tumors, which shows its important role in cellular invasiveness [27].

## 5. Conclusion

Our research shows that the level of autophagy in EM tissues is lowered. Furthermore, different methods of interfering in vitro with the autophagy levels of endometrial CRL-7566 cells demonstrated the important influence autophagy has on the cells' growth. This in turn corroborates the function of autophagy in the proliferation and invasiveness of endometriotic cells. Taken together, this study opens the prospect of elucidating the mechanisms leading to the development of EM, as well as offering new ideas and a theoretical basis for its prevention and treatment.
